# A new synonym-substitution method to enrich the human phenotype ontology

**DOI:** 10.1186/s12859-017-1858-7

**Published:** 2017-10-10

**Authors:** Maria Taboada, Hadriana Rodriguez, Ranga C. Gudivada, Diego Martinez

**Affiliations:** 1 Department of Electronics & Computer Science, University of Santiago de Compostela, Campus Vida, Santiago de Compostela, 15705 Spain; 2 CareCentrix, Hartford, 06103 Conneticut USA; 3 Department of Applied Physics, University of Santiago de Compostela, 15705, Santiago de Compostela, Campus Vida Spain

**Keywords:** Biomedical ontologies, Entity name discovery, Human phenotype ontology, PubMed

## Abstract

**Background:**

Named entity recognition is critical for biomedical text mining, where it is not unusual to find entities labeled by a wide range of different terms. Nowadays, ontologies are one of the crucial enabling technologies in bioinformatics, providing resources for improved natural language processing tasks. However, biomedical ontology-based named entity recognition continues to be a major research problem.

**Results:**

This paper presents an automated synonym-substitution method to enrich the Human Phenotype Ontology (HPO) with new synonyms. The approach is mainly based on both the lexical properties of the terms and the hierarchical structure of the ontology. By scanning the lexical difference between a term and its descendant terms, the method can learn new names and modifiers in order to generate synonyms for the descendant terms. By searching for the exact phrases in MEDLINE, the method can automatically rule out illogical candidate synonyms. In total, 745 new terms were identified. These terms were indirectly evaluated through the concept annotations on a gold standard corpus and also by document retrieval on a collection of abstracts on hereditary diseases. A moderate improvement in the F-measure performance on the gold standard corpus was observed. Additionally, 6% more abstracts on hereditary diseases were retrieved, and this percentage was 33% higher if only the highly informative concepts were considered.

**Conclusions:**

A synonym-substitution procedure that leverages the HPO hierarchical structure works well for a reliable and automatic extension of the terminology. The results show that the generated synonyms have a positive impact on concept recognition, mainly those synonyms corresponding to highly informative HPO terms.

**Electronic supplementary material:**

The online version of this article (10.1186/s12859-017-1858-7) contains supplementary material, which is available to authorized users.

## Background

Named entity recognition has proved very useful in biomedical text mining. Recently, it has been successfully applied to identify entities in cancer research [1], heart disease risk factors in diabetic patients [2], long non-coding RNAs-protein interactions [3] or phenotypic information [4], among others. Biomedical named entity recognizers fall mainly in the broad categories of terminology-based, rule-based, and statistical pattern learning-based approaches [5]. In addition, ontologies have been playing a key role as terminology resources to mine biomedical texts [6]. However, ontology concepts are hard to recognize in free text as their general representation in the ontology is different from their descriptions in text [7].

### Phenotype annotation

Automated analysis of scientific and clinical phenotypes narrated in the literature has gained increasing attention due to the recent progress in using the Human Phenotype Ontology (HPO) to encode phenotypes [8]. In clinical domains, a *phenotype* is a divergence from normal morphology, physiology or behavior [9]. The HPO, which is accessible at www.human-phenotype-ontology.org, contains more than 11,000 concepts designating human phenotypic abnormalities, as well as hierarchical relationships between concepts [10]. The ontology has been primarily developed to deliver a standardized core of human disease manifestations for computational analysis, and it is regularly updated and distributed. Concept recognition using the HPO has immense potential to automatically extract information from large amounts of existing patient records or controlled trials. However, recognizing phenotypes represents a challenge, largely due to the highly lexical and syntactic variability in referring phenotypes in free text [11]. To mitigate the problem, concept recognizers leveraging HPO as a direct target have emerged, such as the Bio-LarK CR [11] or the OBO Annotator [12]. Additionally, some studies have manually extended the HPO in order to ensure accurate annotation [13].

**Fig. 1:**
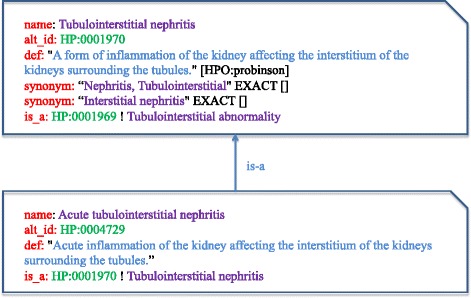
Part of the entries for the term *acute tubulointerstitial nephritis* and the direct ascendant term *tubulointerstitial nephritis*. The HPO terms have a unique identifier, a name, and many of them have textual descriptions and synonyms, that is, words that have the same meaning, or more or less the same meaning as another word, according to Wikipedia. In the format provided by the Open Biomedical Ontology (OBO) Foundry, the type of a synonym may fall into one of the following categories: exact, broad, narrow, and related. The hierarchical relationships between two terms are expressed using the is-a entry

To exemplify the problem, we examined the ten top search results of the term *acute tubulointerstitial nephritis* (HP:0004729) on Google (April 2017). Fig. 1 partially shows the entries for this term and its direct ascendant term in the ontology file. At the time of the test, Google returned five links to web sites relevant to the term *acute interstitial nephritis,* as it recognizes this term as synonymous with the given search term*.* However, *acute interstitial nephritis* could not be recognized using the services provided by the NCBO Annotator [14] and Bio-LarK CR [11],^1^ as the HPO did not include this term as synonym at the time of the study. Additionally, when the search term was entered into PubMed, fewer than 30% of abstracts in MEDLINE relevant to the term were recovered. Hence, new procedures oriented to automatically produce good vocabularies from ontologies are still required for named entity based annotation.

### Techniques to extend biomedical terminologies

Over the years, different approaches have been proposed to extend biomedical terminologies. Interesting synonym-substitution techniques, based on processing word-level terms, have been developed for enhancing the process of concept discovery in the UMLS [15–17] and SNOMED CT [18]. In all these approaches, new synonyms were created from multi-word phrases by replacing one or more words with known synonyms. The latter includes 1) the synonyms of individual words retrieved directly from the terminology, and 2) the terms generated, at an intermediate stage, by removing common subsequences of words shared between two multi-word synonyms existing in the terminology [15–17]. For example, if *kidney biopsy* was synonymous with *renal biopsy*, then dropping the common word *biopsy*, synonymy between *kidney* and *renal* was inferred. A shortcoming with this approach was the generation of millions of candidate synonyms, many of which were not suitable for the clinical domain. In addition, the method did not resolve the homonym problem, as it replaced the synonyms without consideration of the original meaning of the term. Consequently, if a term conveyed two different meanings, then the substitution phase did not resolve which of the two original meanings should be associated with the candidate synonym. Finally, the method generated synonyms without discrimination between different types of specificity (such as, exact, related, etc.) leading to term ambiguity. In order to address the challenge of combinatorial explosion, in [17] two methodological parameters (*maximum number of substitutions per term* and *maximum term length*) were constrained, whereas in [18] other different conditions (*minimal number of hits in the ontology* and *maximum number of synonyms per term*) were imposed.

Another interesting proposal for enriching controlled vocabularies [19] involved extracting a corpus of phrases from MEDLINE and comparing the extracted terms to the concepts in the terminology (in this case, UMLS). The corpus was restricted to those phrases starting with one or several adjectival modifiers. A phrase became a candidate synonym if both the modifiers and the demodified term (i.e., the phrase resulting from removing its adjectival modifiers) were found in the UMLS Metathesaurus. In order to do this, Natural Language Processing (NLP) techniques were required, and the identified problems, such as incorrect identification of part of speech or acronyms, mainly came from the application of these techniques. On the other hand, in [20] the generation of synonyms was done by a rule-based system, which rewrote and suppressed terms based on UMLS properties. In general, rule-based approaches require deeper domain knowledge; they are time consuming, and dependent on lexicon fast updates.

It is worth pointing out that efforts in a similar area, such as ontology mapping, are of a comparable nature. [21] used both the lexico-syntactic properties of the HPO terms and the logical structure of the ontology to discover partial mappings between HPO and SNOMED CT. The authors compared both the lexico-syntactic and logical approaches and concluded that they were complementary to each other. Additionally, [22] proposed a new method to measure lexical regularities in biomedical ontology terms with the aim of discovering new relationships between them.

### Compositionality of the gene ontology and the HPO

Over the past two decades, different studies have examined and leveraged the compositional structure of several biomedical ontologies, among others, the Gene Ontology (GO) and the HPO. It is not uncommon to find GO terms that include its parent terms as proper substrings [23–25]. This property was used to augment the GO itself, with the challenge of refining regulatory relationships recognition from MEDLINE abstracts [26]. Using the compositional nature of the GO, synonymy was inferred by identifying common syntactic patterns within the GO [27]. This method generated synonyms (such as orthographic variants, abbreviations, or chemical products), just as the synonym-substitution techniques [15–18] created new terms at the intermediate step.

A more recent approach [28], also built on the compositional nature of the GO, inferred synonymy by applying a set of syntactic and lexical rules on the constituent terms. This synonym-substitution technique broke down the GO terms into its components parts, and replaced these constituent parts with GO synonyms and derivational variants. Whereas the above-mentioned synonym-substitution techniques [15–18] identified common subsequences of words shared between pairs of known synonyms, [28] applied a set of syntactic rules in order to split up the ontology terms. Additionally, the latter produced intermediate-level synonyms by applying derivational variant generation rules. In order to preserve the quality of GO, irrespective of the technique used, the generated terms must follow established conventions for the expression of concepts. [29] proposed an automated method for ontology quality assurance, which was based on identifying the occurrence of terms expressing similar semantics with different linguistic conventions.

Concerning the HPO, some terms are phrases using a combination of anatomical entities and qualities [30]. This compositional nature has provided the opportunity of logically defining the HPO terms, using the strategy known as *Entity-Quality decomposition*. The strategy was applied for mining skeletal phenotype descriptions from scientific literature [31] and integrating phenotype ontologies across multiple species [32]. Phenotype descriptions show high lexical variability, mainly in qualities. With the aim of improving recall in phenotype concept recognition, [33] proposed to automatically build a dictionary of lexical variants for human phenotype descriptions.

### Specific contribution

In this work, we present a new automated synonym-substitution procedure aimed at enriching the entire HPO with new synonyms. Unlike the techniques described above [15–18], which were mainly based on the lexical properties of the ontology terms, our approach also takes the hierarchical structure of the ontology into account in order to produce synonym-substitution. Furthermore, on the basis that the HPO structure is highly compositional [30–32], we hypothesize that the HPO could be enriched by means of identifying those terms that include descendant terms as proper substrings. However, our method does not break down the terms into its components parts (affected entities and abnormal qualities) [31, 32], but rather it identifies common subsequences of words shared between a term and its descendant terms. This makes it possible to apply the technique to the entire HPO and not restrict it to specific parts, such as musculoskeletal or skeletal phenotypic abnormalities. Furthermore, due to PubMed is an excellent resource providing updated accurate evidence over the use of the terminology by the community, we also hypothesize that validating the existence of the generated synonyms by searching for these exact phrases in MEDLINE can help automatically rule out illogical synonyms. The work has been carried out in the context of the national project OntoPhen, an initiative oriented to provide tools for facilitating the deep phenotyping of the rare disease known as Spinocerebellar ataxia type 36 (SCA36).

## Methods

**Fig. 2:**
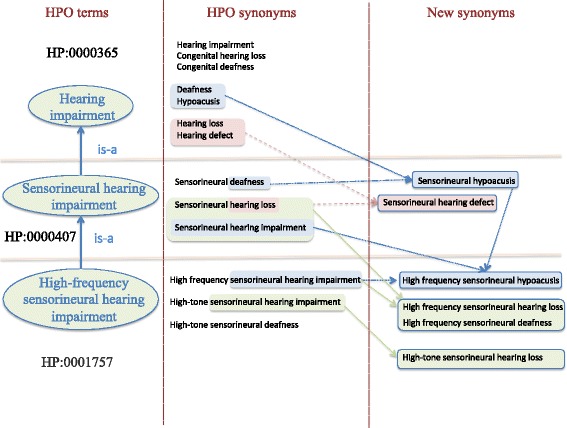
Overall flow of synonym generation

Our synonym-substitution method can be summarized as follows. First, the method rules out redundant synonyms from the point of view of named entity recognition. Then, it recursively identifies all the lexical overlaps in the HPO, that is, all pairs of terms connected by a hierarchical relationship and where the descendant term includes the ascendant term as a proper substring. This step exploits the transitive closure of the HPO hierarchical relationships. Subsequently, for each descendant term in every lexical overlap, the method generates new synonyms by replacing, in the descendant term, the overlapped words with known synonyms of the ascendant term. Finally, it searches the exact phrases of the generated synonyms in MEDLINE, and it rules out the ones for which no result were retrieved. Additionally, since the HPO provides different levels of relatedness in synonymy, this aspect is propagated through the generated synonyms. Fig. 2 depicts the flow of synonym generation.

### Ruling out redundant synonyms

**Fig. 3:**
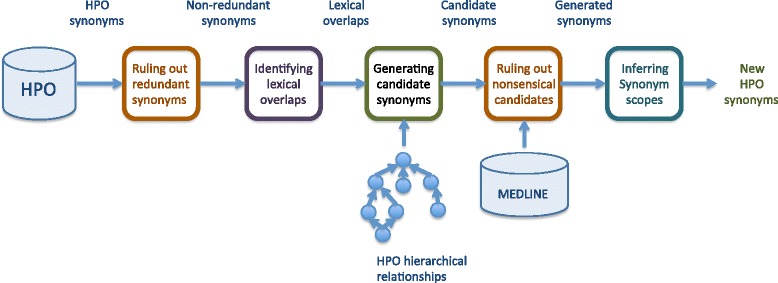
Example of synonymy generated by our method. On the left side, a very small excerpt of the HPO hierarchy for *hearing impairment* (HP:0000365) is shown. On the middle side, for each HPO class, part of the current synonym set is shown. The lexical differences between some terms and its descendant terms are highlighted in color. Different lexical overlaps are underlined in different colors, only to make it easier to identify them in the figure. On the right side, some new synonyms generated by our method are displayed. The arrows show the origin of the new synonyms

We detected that there were synonyms accommodating other synonyms of the same term as proper substrings, leading to degraded performance of our method. For example, in Fig. 3, *congenital hearing loss* includes the string *hearing loss*. Both of them are synonyms of the concept HP:0000365 (*Hearing impairment*). Generally, a concept recognizer identifying *congenital hearing loss* will also recognize *hearing loss*. Thus, *congenital hearing loss* can be considered as a redundant synonym from the point of view of concept recognition. Hence, we decided to remove all redundant synonyms from the HPO.

### Identifying lexical overlaps in the HPO

Although the notion of lexical overlap applies to a pair of arbitrary terms where one of them encompasses the other one as a proper substring, we chose to restrict its application to our purpose, i.e. to a pair of terms connected by a hierarchical relationship. For example, in Fig. 3, lexical overlap exists between *hearing loss* and *sensorineural hearing loss*. In short, lexical overlaps are the reiterated largest fragments of text occurring in the strings of two terms (or synonyms) with a hierarchical relationship between them.

**Fig. 4:**
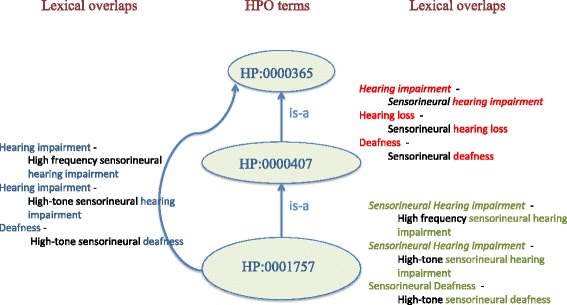
Example of lexical overlaps identified by our method

For each top-level phenotype category, this stage extracted all pairs of HPO terms that were lexical overlaps, from the root node of the category to the leaf nodes. Note that the transitive closure of the HPO hierarchical relationships was exploited. In simple terms, for each pair of unique terms that were directly or indirectly connected between them through a hierarchical relationship, the method checked for all string matches between their synonyms. For example, for the pair of unique terms HP:0000365 and HP:0000407, three lexical overlaps were identified (upper right part of Fig. 4); for HP:0000365 and HP:0001757, another three lexical overlaps are identified (left part of Fig. 4); and for HP:0000407 and HP:0001757, three more lexical overlaps exist (bottom right part of Fig. 4).

### Generating new synonyms recursively

For each identified lexical overlap, the method recursively generated new synonyms for each descendant term in the overlap. The generation of new synonyms was carried out by synonym-substitution (i.e., by replacing the overlapped substring in the descendant terms with known synonyms for the ancestor terms). For example, replacing *hearing loss* in *sensorineural hearing loss* (upper right part of Fig. 4) with the synonym *hearing defect*, the synonym *sensorineural hearing defect* was generated (right part of Fig. 3). Similarly, replacing *hearing impairment* in *high-tone sensorineural hearing impairment* (left part of Fig. 4) with the synonym *hearing loss*, *sensorineural hearing defect* was generated (right part of Fig. 3).

### Ruling out the nonsensical synonyms

The preceding steps did not ensure that the generated synonyms were syntactically correct or widely accepted in the biomedical domain. The use of nonsensical terms would degrade the performance of named entity recognition. In order to solve the problem, we decided to rule out the nonsensical candidate synonyms. The large number of publications in MEDLINE, daily updating, and easily accessible through PubMed,^2^ made it suitable for verifying the terminology quickly, effectively and precisely. Our assumption was that terms not included in any publication in MEDLINE were incorrect. With this in mind, the method searched for the exact phrases in MEDLINE^3^ (only in the title and abstract fields). For example, the method did not find the exact phrase “*high frequency sensorineural hypoacusis”* in MEDLINE, so it ruled out the synonym.

### Inferring types of synonyms

**Fig. 5:**
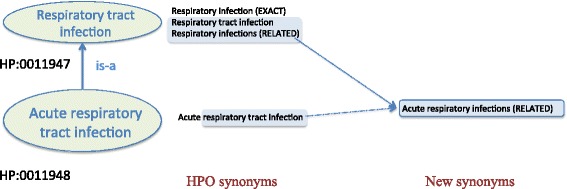
Example of the synonym type inferred by our method. On the left side of the figure, a subsumption relationship between *acute respiratory tract infection* and *respiratory tract infection* is shown. The first term includes the second one as a proper string. On the middle side, for these two terms, the synonym set is shown. The synonym *Respiratory infections* was used for replacing the overlapped string. As the type of the substituted synonym was *related*, the method inferred the type *related* for the generated synonym *Acute respiratory infections*, which is displayed on the right side

For each generated synonym, the method inferred its type (or scope) from the type of both the pair of terms in the lexical overlap and the synonym used for substitution. Specifically, the method inferred the most restrictive type of these terms. For example, in Fig. 5, the parent term was included in the descendant term as a proper string, so the method identified a lexical overlap between them. Then, the method replaced the overlapped string *respiratory tract infection* with the synonym *Respiratory infections*, generating the new term *acute respiratory infections.* Next, the method inferred the type *related*, as the type of *acute respiratory infections* was “*related”*.

### Evaluation procedure

We evaluated the research value of the generated synonyms extrinsically by measuring their contribution to the performance of a concept recognition system. Specifically, we assessed the performance of two aspects: concept annotation and document retrieval. To that end, two types of different corpora were used in the evaluation. The first one is a corpus of 228 abstracts [11] cited by the Online Mendelian Inheritance in Man (OMIM) database [34] and manually annotated by a team of three experts. It includes 1933 concept annotations, which cover 460 different HPO concepts (over 4% of all unique terms). Although the set of annotations is reduced in relation to the size of the HPO, there is no another corpus with text-level HPO annotations. This corpus was used as a gold standard for evaluating the contribution of the new terms to measure the performance of concept annotation.

At the moment, the HPO development not only depends on OMIM but several other resources, such as the medical literature. Hence, the gold standard might not cover all relevant terminology. Therefore, we decided to measure the contribution of the new synonyms towards the performance of document retrieval. For this purpose, we prepared a collection of abstracts from MEDLINE. As HPO is primarily used in hereditary disease annotations for allowing large –scale computational studies of the human phenome, a Pubmed search was performed with the keyword “*hereditary disease”*. In total, 580,308 abstracts were utilized for our evaluation. Additionally, we calculated the information content (IC) of the unique HPO terms, based on the curated annotations provided by the HPO consortium [4]. The IC is quantified as the negative log-likelihood function [35]:

$$ IC=-{log}_{10}\ p(t) $$

In our work, *p(t)* was the probability of appearing the term t in the curated annotations.

$$ p:T\to \left[0,1\right] $$

with T the set of the unique HPO terms. A term with a lower IC score means that it is being used to annotate many human hereditary syndromes and it should occur frequently in the literature. From [28], terms with a higher IC score are less likely to appear in texts, and hence more informative. Therefore, methods generating synonyms with a higher IC score will have a major impact at the concept recognition task and so, document retrieval.

The evaluation process used the OBO Annotator [12], a concept recognizer oriented to perform automatic annotation of phenotypes based on the HPO. The following provides a brief overview of how the OBO Annotator works. First, it splits the input text into smaller chunks, which are preprocessed and then looked up in a dictionary preprocessed from the OBO ontology. The preprocessing step removes common words and punctuation marks. Second, it applies stemming and permutations of the word order, which generates term variants. More detailed annotations are provided over more general ones, when overlapping annotations exist.

The evaluation procedure consisted of creating two dictionaries, the first one uses the HPO itself as the synonym repository and the second one is created by adding new synonyms to the first dictionary. Later, the OBO Annotator was run once using each dictionary. We report precision, recall and F-measure from the evaluation on concept annotation, and percent change in annotations from the evaluation on document retrieval.

## Results

Our experiments leveraged the HPO data version released on 2016–01-13, MEDLINE was accessed via PubMed on 2016–05-11 in order to filter the generated synonyms and on 2017–05-03 to generate the collection for evaluation.

### Lexical overlaps of the HPO ontology

Each term in the HPO has a unique identifier, a name and a list of synonyms. Table 1 shows the main properties used as metrics for the lexical overlaps in the HPO. In our experiments, the ontology in OBO format contained 11,004 unique terms. After removing 57 obsolete terms, 10,947 unique terms were taken into account. In total, 18,385 synonyms were distributed into 23 main categories represented by taxonomies. On average, there were 1.68 synonyms per each unique term. In addition, the number of tokens, that is, the text chunk into which a synonym can be divided using a white space character as a delimiter, ranged from 1 to 12. However, 86% of the synonyms contained at most 4 tokens.

**Table 1:** Metrics used for the lexical overlaps in HPO

Property	Number
Total number of non obsolete terms	10,947
Total number of synonyms (including term names)	18,385
Total number of synonyms involving other synonyms of the same term as substring	529
Total number of synonyms	17,856
Number of synonyms per term/concept	1.68
Total number of identified different lexical overlaps	1285

Overall, 529 synonyms involved other synonyms of the same term as proper substrings. After removal, 17,856 synonyms were taken into account. The total number of unique lexical overlaps detected in HPO was 1285, which was almost 12% of the total number of unique terms and 7% of the total number of synonyms.

In order to count the total unique lexical overlaps, we first preprocessed them by following the steps below.

- Hyphenated words were broken into its constituent words. For example, “criss-cross atrioventricular valves” was converted into “criss cross atrioventricular valves”.
- Tokens in brackets were not counted, as generally they are clarifications or acronyms, and they are not suitable for text mining solutions. For example, “thyroid stimulating hormone receptor (tshr) defect” was considered to have five tokens.

**Fig. 6:**
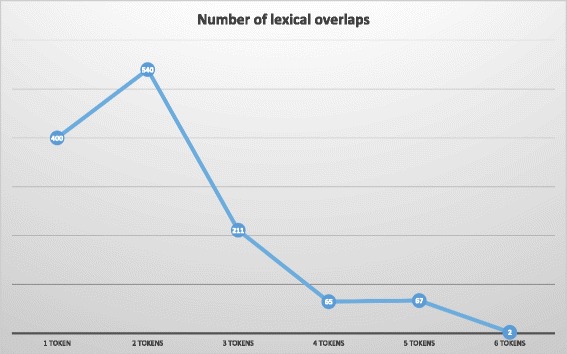
Number of unique lexical overlaps in terms of its number of tokens

This preprocessing stage was the only part of our method that involved the specialized syntax of the ontology. In Fig. 6, we can see the number of unique lexical overlaps broken down by the number of tokens they included. As might be expected, as the number of tokens increased, the number of lexical overlaps decreased, except in those cases for overlaps with two tokens: 540 overlaps with two tokens against 400 overlaps with only one token. The identified lexical overlaps are provided as supplementary information with this article (Additional file 1).

### Generating new synonyms for the HPO ontology

The total number of generated synonyms by substitution was 121,594 (see Table 2), including 115,630 synonyms already existing in the HPO. All such duplicated synonyms were removed. The set difference A/B = {x: x ∈ A and x ∉ B} included 5964 synonyms representing 32% of total synonyms in the HPO.

**Table 2:** Number of synonyms generated by the method

Method for generating synonyms	Number of candidate synonyms
Synonym-substitution procedure in lexical overlaps (A)	121,594
Intersection of the set A and the original synonyms in the ontology (B)	115,630
Set difference A/B	5964

### Ruling out the nonsensical synonyms

Of the total 5964 candidate synonyms, only 745 of them were found in MEDLINE by PubMed, when exact phrases were searched (see Additional file 2). The generated synonyms cover 488 unique HPO terms. Concerning the synonym type, 67% of new synonyms were exact, 21% were related, and 12% were synonyms with no relatedness. The latter comes from HPO terms from which there was no information about relatedness.

After ruling out the nonsensical synonyms, the total number of new synonyms was 7% of total unique terms, 4% of total synonyms and 58% of total lexical overlaps. If compared, the total number of newly identified synonyms (745) to the total lexical overlaps (1285), the proportion was significantly higher (58%).

### Evaluation on concept annotation

Table 3 shows the results of the methods called *baseline* and *synonym-substitution in lexical overlaps*. The first method incorporated the data dictionary created from the HPO and the second method was developed on extending the first dictionary from the generated synyonyms. The results show a modest increase in precision (0.02) and recall (0.04).

**Table 3:** Results for the two methods on the corpus, using the Obo Annotator, in terms of precision, recall and F-measure

Method	#Annotations	# Terms	Precision	Recall	F-measure
Baseline	1232	292	0,69	0,44	0,54
Synonym-substitution in lexical overlaps	1253	308	0,71	0,48	0,57

We now examine how many generated synonyms contribute to the increase of performance on the gold standard. In total, our method generated 745 synonyms covering 488 unique HPO terms, although only 36 of them were covered by the gold standard annotations. In other words, only 8% of the unique terms annotating the gold standard were terms with new synonyms. Hence, the results suggest that the modest increase in performance comes from a low coverage of terms with new synonyms in the gold standard.

### Information content (IC) of terms

At the time of the evaluation (April 2017), the HPO consortium provided 129,373 annotations of HPO terms to 9557 human hereditary syndromes listed in OMIM, Orphanet and DECIPHER. These annotations covered 8237 (75%) unique HPO terms. The IC scores for all terms in the HPO are depicted in Table 4. These scores ranged in the interval (0–4).

**Table 4:** Number of the unique HPO terms and number of unique terms for the new synonyms classified by information content

IC	# of the unique HPO terms	% of unique HPO terms	# of the unique terms for the generated synonyms	% of unique terms for the generated synonyms
(0,1)	269	2%	1	0%
[1,2)	773	7%	37	8%
[2,3)	2227	20%	154	32%
[3,4)	4968	45%	232	48%
undefined	2710	25%	64	13%
Total	10,947	100%	488	100%

terms that were not included into the curated annotations were classified as *undefined*. As we can see in Table 4, 25% of HPO terms are *undefined*, whereas 65% of terms have a score higher than 2. With regard to the generated synonyms (745), they correspond to 488 unique HPO terms, where 80% of them have a score higher than 2. Hence, a high percentage of the generated synonyms are highly informative, and so, they are expected to have a positive impact on concept recognition.

### Evaluation on the collection of abstracts

We evaluated the impact of the generated synonyms by counting the number of abstracts whereas at least one unique term was recognized. Statistics for both the terms using the HPO (baseline method) and the extended HPO with the generated synonyms can be seen in Table 5. As the difference between the annotations of both procedures was in the 488 unique terms corresponding to the 745 generated synonyms, we show the increasing rate of annotated abstracts with respect to these 488 unique terms. Results are disaggregated by IC and number of abstracts annotated per term. Overall, 142,043 (24%) abstracts were annotated with some of the 488 unique terms. Of that total, 134,367 abstracts were annotated with the baseline method; and hence, 6% of the 142,043 annotated abstracts were due to the generated synonyms (see the last row of the Table 5).

**Table 5:** Results for the two methods on the abstract collection on hereditary diseases, using the Obo Annotator. They are expressed in terms of the number annotated abstracts by each method. The increase rate is percent change in total annotations. Additionally, theresults are disaggregated by IC and number of abstracts annotated per term

IC	# Abstracts per term	# Terms	% Terms	# Annotated Abstracts (Baseline)	# Annotated Abstracts (with generated synonyms)	Increase rate of annotated abstracts
[0,1)	>1000	0		0	0	0
	[100–1000)	0		0	0	
	(0–100)	1	1	2	2	100
	Total	1	1	2	2	100
[1,2)	>1000	3	8	7061	7126	1
	[100–1000)	18	49	6799	7648	12
	(0–100)	16	43	308	449	46
	Total	37	100	14,168	15,223	7
[2,3)	> 1000	10	6	90,065	90,175	0
	[100–1000)	60	39	17,265	19,655	14
	(0–100)	84	55	3192	4007	26
	Total	154	100	110,522	113,837	3
[3,4)	> 1000	0	0	0	0	0
	[100–1000)	32	14	7366	8377	14
	(0–100)	200	86	2004	4041	102
	Total	232	100	9370	12,418	33
undefined	> 1000	0		0	0	
	[100–1000)	0		0	0	
	(0–100)	64	100	305	561	84
	Total	64	100	305	561	84
Total	> 1000	13	3	97,126	97,301	0
	[100–1000)	110	23	31,430	35,680	14
	(0–100)	365	75	5811	9060	56
	Total	488	100	134,367	142,041	6

Of the 488 unique terms, 13 (3%) terms annotated more than 1000 abstracts (row “Total” and “>1000”, highlighted in light brown in Table 5). These terms correspond to IC values lower than 3 (see rest of the rows highlighted in light brown in Table 5). The generated synonyms for these terms annotated only in the ranges of 0% and 1% of abstracts. An example is the term *Atopic dermatitis* (HP:0001047), which annotated more than 1000 abstracts, and the generated synonym *Atopic skin inflammation*, only annotated 18 abstracts.

In total, 110 (23%) terms annotated a number of abstracts in the range between 100 and 1000 (rows highlighted in green in Table 5). More than 50 % of these terms had IC values between 2 and 3, and they annotated 14% of abstracts. An example is the term *Progressive hearing impairment* (HP:0001730), which annotated over 110 abstracts, and the generated synonym *Progressive deafness*, which annotated 23 more abstracts.

Finally, 365 (75%) terms annotated a number of abstracts in the range between 1 and 100 (rows highlighted in blue in Table 5). More than 70 % of these terms had IC values higher than 3 or they were undefined, and they annotated 56% of abstracts. If we observe the total for IC values higher than 3, 33% of abstracts were annotated. An example is the term *high-output congestive heart failure* (HP:0001722), which annotated over five abstracts, and the generated synonym *High-output cardiac failure*, which annotated 35 more abstracts.

## Discussion

### Lexical overlaps in the HPO ontology

The proposed analysis of lexical overlaps between pairs of terms linked by HPO taxonomic relationships can be viewed as a new method to quantitatively measure how the ontology is following the systematic naming convention; specially when using genus-differentia style names [36], that is, when term names reflect differences between the term and its parent term. We can interpret the results of Table 2 as a high degree of using that convention, as from all potential synonyms that could be generated from the hierarchical relationships in the ontology (121,594), 95% of these (115,630) are included into the ontology. Note that these numbers include repetitions.

### Evaluation on concept annotation

A proper assessment of the results is particularly difficult. In general, using a gold standard is the most appropriate technique for doing so. However, the results of the evaluation show only a modest increase in the performance of concept annotation. This is due to two aspects. First, the use of a limited number of annotated abstracts does not provide the ability to evaluate all the generated terminology, but only a reduced part. It must be noted in this context that our synonym-substitution method aided in the recognition of 15 more abstracts (7% of the total abstracts) for a total of 16 new unique terms. This represents an increase of 44% of the unique HPO terms covered by both the gold standard and the generated synonyms. Second, the gold standard does not cover all relevant terminology in the HPO. In fact, the manual annotations included in the gold standard only covered 8% of the unique terms related to new synonyms.

Some examples of the generated synonyms improving performance on the corpus are shown in Table 6. These synonyms are in fact lexical variations of the existing HPO terms. The results suggest that their use improves the performance of concept annotation when compared to only using the ontology itself as the synonym repository.

**Table 6:** Example of five new synonyms improving the performance on the corpus. By lexical difference between the term name and the ascendant term, the method learns new names (shown as ‘generated synonyms’). The column ‘level in the hierarchy’ shows if the hierarchical relationship between the term and the ascendant term is direct (first level) or indirect (second level and so on)

HPO ID	Term name	Ascendant term name	Ascendant synonym	Level in the hierarchy	Generated synonym
HP:0100019	Cortical cataract	Cataract	Lens opacities	Second	Cortical lens opacities
HP:0008069	Neoplam of the skin	Neoplasm	Cancer	Second	Cancer of the skin
HP:0012715	Profound hearing impairment	Hearing impairment	Hearing loss	First	Profound hearing loss
HP:0007270	Atypical absence seizures	Seizures	Epilepsy	Fourth	Atypical absence epilepsy
0000122	Unilateral renal aplasia	Renal agenesis	Renal aplasia	First	Unilateral renal agenesis

### Evaluation on the collection of abstracts

As can be seen in Table 5, both the terms with the highest IC (greater than 3) and the terms classified as *undefined* show the largest rise in number of annotated abstracts. This confirms that the synonym-substitution procedure leads to lexical variations that can help to recognize a greater number of abstracts containing more specific terms. The difference in the number of annotated abstracts is less important for the terms with lower IC; specially for those terms annotating a number of abstracts higher than 100.

With the aim of drawing further conclusions, we revised a random sample of 2% of abstracts annotated with the generated synonyms. We found the following results. First, some generated synonyms were morphological variations of the HPO synonyms, such as *respiratory recurrent infections*. As the OBO Annotator generates variants of the ontology terms, the inclusion of these morphological variations did not bring about any changes in number of annotated abstracts. In total, we detected that 14% of generated synonyms were morphological variations. However, the addition of these morphological variations could be helpful when using concept recognizers other than the OBO Annotator. Second, some generated synonyms were included in other HPO synonyms as proper substrings. For example, the method generated the new synonym *elbow joint dislocation* for the HPO term *elbow dislocation*. In cases like this, the inclusion of these synonyms did not involve a change in the number of annotated abstracts. Third, we detected some unusual errors in our method. An example is the synonym *anterior spinal fusion.* This term was not ruled out through the search in Pubmed, as it appears as part of the larger string *anterior spinal fusion surgery* in MEDLINE. However, this type of errors was extremely rare.

Finally, a potential drawback of our evaluation is that, we conducted this research 16 months after we firstly accessed the HPO. In order to address this limitation, we compared the release used in our work (January 13, 2016) and the version later from April 13, 2017. In total, the newest version provided 1222 more synonyms (including term names and excluding obsolete terms) than the version used for this study. Furthermore, it provided only 20 (3%) of the synonyms generated by our method. The list of these synonyms is provided as supplementary information with this article (Additional file 3).

### Future work

In the future, we plan to extend our synonym-substitution procedure by identifying lexical regularities among concepts linked by some logical axiom, not only hierarchical axioms. Furthermore, one limitation of our method is the need to identify lexical overlaps. An alternative to solve this problem is to initially increase the number of synonyms only for the roots of the hierarchies in the ontology. In the near future, our intention is to add this extra step to our method. Finally, we think that our method could be adapted to automatically select the most appropriate synonyms of the ontology to concept recognition tasks. The method would compute, for each concept, the central term (that is, the term at a minimal average distance of any term in the concept), so the rest of the terms would be ranked for the minimal distance to the central term. Thus, the central term would become the preferred term for concept recognition tasks.

Finally, we think that we could automatically extend our proposal according to a similar principle as [13] did before, but limited to the HPO concepts. For example, for the HPO concept *interstitial nephritis*, we could search for all words (excluding stop words) that are near to it (i.e., collocates). In this case, we could identify the new modifier *granulomatous* for the term *interstitial nephritis,* and generate the new term *granulomatous interstitial nephritis.*

## Conclusions

The efficacy of the ontology-based approach for concept recognition relies on the coverage of synonyms for the specific domain and how well these synonyms are appropriate for natural language processing. However, ontologies are not designed specifically to be the lexical basis for text mining or name recognition systems, so the performance of ontology-based approaches is lower than required. This research has showed that it is possible to automatically recognize new lexical variations for the HPO synonyms, using both the lexical and logical properties of the ontology. In addition, the search engine Pubmed provided an effective method to filter nonsensical synonyms. We showed that the generated synonyms have a positive impact on concept recognition, mainly the ones corresponding to highly informative HPO concepts.

## Additional files

Additional file 1: Supplementary information: Full list of resulting lexical overlaps. (TXT 25 kb)

Additional file 2: Supplementary information: Full list of new synonyms, with information about the normalized semantic distance and trends. (CSV 62 kb)

Additional file 3: Supplementary information: Full list of the 20 synonyms generated by our method from the release of the HPO from January 13, 2016 and provided by the version of the HPO from April 13, 2017. (CSV 1 kb)

## Abbreviations

HPO: Human Phenotype Ontology
NSD: Normalized semantic distance
OBO: Open Biomedical Ontology
OMIM: Online Mendelian Inheritance in Man
PT: Preferred terms
SCA36: Spinocerebellar ataxia type 36
UF: Usage frequency

## Footnotes

1 The Monarch HPO Annotator http://jannotator.monarchinitiative.org/#/.

2
http://www.ncbi.nlm.nih.gov/pubmed

3
http://www.nlm.nih.gov/bsd/pmresources.html
